# Narrative-based learning for person-centred healthcare: the Caring Stories learning framework

**DOI:** 10.1136/medhum-2022-012530

**Published:** 2023-05-19

**Authors:** Laura Mazzoli Smith, Feliciano Villar, Sonja Wendel

**Affiliations:** 1 School of Education, Durham University, Durham, UK; 2 Department of Cognition, Development and Educational Psychology, University of Barcelona, Barcelona, Catalunya, Spain; 3 Erasmus School of Economics, Erasmus Universiteit Rotterdam, Rotterdam, Zuid-Holland, Netherlands

**Keywords:** patient narratives, narrative medicine, Care of the elderly, health care education, inter-professional education

## Abstract

This paper describes the learning framework for an innovative narrative-based training platform for healthcare professionals based on older patients’ narratives. The aim of Caring Stories is to place patients’ desires and needs at the heart of healthcare and by doing so to promote person-centred care (PCC). It is argued that this narrative-based approach to training in healthcare education will provide professionals from different fields with competencies to better understand how to interpret the lifeworlds of older people, as well as facilitate better communication and navigation through increasingly complex care trajectories. The spiral learning framework supports narrative-based training to be accessible to a broad range of healthcare practitioners. We suggest this is a theoretically sophisticated methodology for training diverse healthcare professionals in PCC, alongside core tenets of narrative medicine, with applicability beyond the patient group it was designed for. The learning framework takes into account professionals’ mindsets and draws on the epistemic tenets of pragmatism to support interprofessional education. Being informed by narrative pedagogy, narrative inquiry, and expansive learning and transformative learning theories, ensures that a robust pedagogical foundation underpins the learning framework. The paper sets out the conceptual ideas about narrative that we argue should be more widely understood in the broad body of work that draws on patient narratives in healthcare education, alongside the learning theories that best support this framing of narrative. We suggest that this conceptual framework has value with respect to helping to disseminate the ways in which narrative is most usefully conceptualised in healthcare education when we seek to foster routes to bring practitioners closer to the lifeworlds of their patients. This conceptual framework is therefore generic with respect to being a synthesis of the critical orientations to narrative that are important in healthcare education, then adaptable to different contexts with different patient narratives.

## Introduction

This paper outlines an innovative, narrative-based learning framework for diverse healthcare professionals, based on older patients’ first person narratives. The training platform, Caring Stories, was developed by an international consortium funded by EIT Health.1 The main aim is to place patients’ desires and needs at the heart of healthcare, to promote person-centred care (henceforth PCC). Inclusivity, in terms of a training accessible to healthcare practitioners from any specialism that works to support the care of older patients, was a key consideration. A narrative-based approach to training was chosen, with a particular, and we argue here, well-developed conceptualisation of narrative, for a learning platform, that engages with the complex and contested literature in cognate fields. It is argued that this narrative-based approach to training in healthcare education will provide diverse professionals with competencies to better understand how to interpret the lifeworlds of older patients, and skills to facilitate better communication and navigation through increasingly complex care trajectories and the complexity of ordinary lives. Caring Stories can therefore be said to be a response to the need for a theoretically and pedagogically well-informed learning framework to support the humanistic aims of narrative medicine and PCC.

Conceptually the learning framework is situated in a pragmatist epistemological orientation and draws on narrative pedagogy, narrative inquiry, and expansive and transformative learning theories, as well as key debates in medical humanities concerned with criticality in use of patient narratives in healthcare education. The core ideas from these fields that structure the training are outlined below, in what is primarily a conceptual paper, explicating the distillation of a plurality of ideas, which have been developed over a number of years of the funded grant for this particular training platform. The result, Caring Stories, is currently being trialled with practitioners in diverse settings, such as care homes, council social services and hospitals in the UK, the Netherlands and Spain and a forthcoming publication will report on these. In this paper we set out the conceptual ideas about narrative in particular, that we argue should be more widely understood in the broad body of work that draws on patient narratives in medical humanities and healthcare education, alongside the learning theories that best support this framing of narrative. This conceptual framework functions as a synthesis of what we suggest are critical orientations to narrative that should be foregrounded, from a number of disciplinary perspectives, which can then be adapted and developed in different learning contexts and which we set out in this paper. Caring Stories consists of a series of 40 exercises linked to six sequential learning modules that open up and interrogate the various aspects of what it means to position narrative as a tool, where the narrative encounter is what situates meaning. We scaffold these interpretative exercises through different ways into narrative knowledge, broadly aimed at pluralist interpretation of narratives through content, structure and interpersonal dialogue, in order to foster the dispositions that underpin the PCC paradigm.

It could be said that there is consensus in terms of the importance of PCC in diverse healthcare contexts, but as yet, relatively little consensus with respect to how best to foster this (Britten *et al*. 2020; Harding, Wait, and Scrutton 2015). Nor is there said to be a clear methodology for narrative medicine (Barber and Moreno-Leguizamon 2017; Fioretti *et al.* 2016; Wieżel *et al*. 2017), which links with some of the aims of PCC and which this learning framework could also be drawn on to foster. The argument in this paper is that training for these concepts is well supported through the use of narrative-based learning, but that this should be better conceptualised theoretically in such a way as to take account of epistemological debates in the field of narrative medicine and medical humanities and linked to learning theory. It is the integration of these concerns into the learning framework itself, which we suggest is novel here and yet the resulting training remains inclusive and accessible to practitioners with little or no grounding in the humanities, which must be at the heart of any aspiration for a paradigm shift to PCC. There is a generic quality to the learning framework, which we explicate here in terms of the underlying concepts, so that it can be adapted to any professional context and geographical location, in part through the use of locally derived narratives but also because of its theoretical foundations. The Caring Stories learning framework takes into account professionals’ mindsets and draws on the epistemic tenets of pragmatism to also support interprofessional learning. Being informed by narrative pedagogy, narrative inquiry, and expansive learning and transformative learning theories, ensures a robust conceptual foundation that underpins the pedagogy in a six module spiral learning framework. The framework draws much from wide debates in narrative inquiry and contributes to, rather than sits apart from, the seminal work of narrative medicine (Charon 2008) in bringing a primarily pedagogical approach to how to support the learning of the healthcare professional whose own epistemic orientation in the complex knowledges of healthcare has to be the foundation of learning.

Caring Stories was developed as a training platform for healthcare practitioners working with older patients and the impetus for this is clear. Improvements in healthcare and lifestyle mean that the population is getting older: the ratio of people aged over 65 years for every 1000 people aged 16–64 years (known as the *old age dependency ratio*) is increasing across all European countries. The heterogeneity among older people is enormous, ranging from healthy, participative and active older citizens to others that are living with dementia or other severe conditions that challenge their agency and autonomy. As well as health and autonomy, factors such as gender (Catlett 2022), cultural and ethnic origin (eg, Lavin and Park 2014), or socioeconomic and educational status (eg, Wang and Hulme 2021) contribute greatly to increase diversity. Consequently, priorities in care should follow suit in terms of this diversity. Moreover most older patients, and particularly those who are better educated, if given the opportunity, may want to participate in care decisions and have some control over care choices (Bynum *et al*. 2014; Chiu *et al*. 2016). In care, we need to train professionals to accommodate this heterogeneity and at the same time help them in making choices for the care they provide according to the priorities of older patients, yet therein additional challenges lie. Underwood argues that older persons belong to a distinct social group distinguished by their age, and language used is adapted towards, ‘topical emphasis on the past [… ], pragmatic interpretations of reminiscence, narrative contextualisation, explicit reference to remembering and forgetfulness, and vagueness’ (Underwood (2010, 164)). Villar and Serrat (2017) discuss the problematics of the maintenance of narrative identity in the context of conditions such as dementia, life changes such as entering institutional care, the complexity of care needs in the face of comorbidity and terminal conditions, and the dominant Western meta-narrative of decline in older age (Jenkins 2017). Add to this the debate about narrative identity in relation to ageing and later life (Kenyon, Bohlmeijer, and Randall (2010); de Medeiros 2013) and we see the challenge for any training that seeks to situate the narratives of older patients at its heart.

## Fostering PCC

While the Caring Stories training platform was developed with the healthcare of older patients in mind and this is the context in which it came into being, we are suggesting here that the generic learning framework at its heart can be used in more generalised healthcare education for PCC and in learning aimed to foster models related to PCC. PCC itself is an imprecise term, often used as a collective term incorporating patient-centred care and citizen-centred care, each with their own interpretations (Wilberforce *et al*. 2017). In this paper, we use it as an ‘umbrella’ concept including also other different, but closely related approaches, such as relationship-centred care (Nolan, Keady, and Aveyard 2001, Soklaridis *et al*. 2016) or even, although less used in gerontological contexts, family centred care (Shields 2015). In its diverse conceptualisations, PCC has been adopted as healthcare policy across the globe under its various interpretations (see World Health Organisation 2007; Cooper and Spencer-Dawe 2006; Care Quality Commission 2016, 2017a, b; O’Dwyer 2013 Currie *et al*. 2015; Mckeown *et al*. 2014; Santana *et al*. 2018). In the development of the Caring Stories training platform, we commence from the WHO model ‘in which individuals, families and communities are served by and are able to participate in trusted health systems that respond to their needs in humane and holistic ways’ (World Health Organisation 2007, 7). In addition we work towards an interpersonal orientation:

patient and client-centred care [implies] an individualized therapeutic relationship, whereas PCC attempts to build upon a more interpersonal relationship. [As a] shift is envisioned from a paternalistic biomedical tradition where healthcare experts are omniscient decision-makers to a more humanistic, dialogic and collaborative relationship where lay people in need of medical care are still recognized as resourceful and capable. (Naldemirci *et al*. 2018, 56)

It is widely suggested that this requires changes in established organisational structures and practices towards: understanding the person; engaging the person in decision-making; promoting the care relationship (Wilberforce *et al*. 2017). PCC can be said to depend on an understanding of health outcomes that are broader than the treatment of disease, but rather predicated on addressing illness, ‘the ‘what it is like’ qualitative dimension as it is experienced and made meaningful by the ill person’ (Carel 2016, 3). Carel describes illness as bringing about an existential transformation and as such should be viewed as important as the experience of disease. Most relevant here is that the practical usefulness of doing so through uncovering characteristics of illness, enables a shared world of meaning, which ‘can improve patient-clinician communication, increase patient compliance and trust, assist in medical teaching and training’ (2016, 19). [Bibr R97]A synthesis of the literature across general medical care, nursing, dementia care, social work and rehabilitation settings, lists 12 attributes for PCC under three thematic headings:

1. *Understanding the person*: Understands the personal experience of illness/disability; knows the different dimensions of life requiring support; understands the person’s values and preferences in care; knows what is important to the person’s identity and well-being;
2. *Engagement in decision-making*: Person is involved in the decision-making processes; person’s wishes shape decisions and care plans; flexible care services tailored to individual preferences; information and options are shared in a clear format;
3. *Promoting the care relationship*: Friendly, caring and respectful interactions; continuity and coordination in care relationships; positive attitude to person’s capabilities and roles; reciprocity in care relationship.

While much recent literature focuses on why we need to move to a more PCC model, there is little information on how to do this (Britten *et al.* 2020, 370). In a report by the Health Policy Partnership, Harding *et al*. (2015) conclude that the implementation of PCC in the mainstream was tentative. Their demarcation of the three conceptual pillars of PCC dovetails with that of Wilberforce *et al*. (2017); an overarching group of concepts designed to focus care on patients’ needs and circumstances; emphasis on personhood, rooted in the philosophy of people as persons in contexts in their own social worlds; and the importance of partnership between patient and practitioner, which can only be achieved by symbiosis and the sharing of knowledge and expertise within a therapeutic alliance. There are two core issues that we can distil from these conceptual pillars of PCC for the development of a narrative-based healthcare education. Firstly an orientation to the patient in their sociocultural context and in their own lifeworlds, that is the lived world as experienced in everyday situations and relations (van Manen 1990).

Secondly the centrality of the relational and shared knowledge, and interpretation of care needs in and through communication. Communication must therefore be conceived as effected in social interaction, relationally, embodied and at multiple levels of awareness (Smith 2007).

There are both ontological and epistemological issues at stake here, which should then inform learning methodology and pedagogy with these conceptual pillars in mind. We move on to consider narrative in both medical and healthcare education with PCC aims and how best to conceptualise narrative in order to support these.

## Narrative in medicine and healthcare training

Medicine and healthcare education employ narrative in multiple ways to complement traditional biomedical practices; for example, narratives are used in the investigation, diagnosis of illness and treatment (Boudreau, Cassell, and Fuks 2009; Hicks *et al.* 2012), in narrative therapy (Bhar 2015; Kropf 1998), and in day-to-day operations in care settings (Britten *et al*. 2017; Moore *et al*. 2017; Naldemirci *et al*. 2018; Nolan *et al*. 2004) or in the community (d’Araújo *et al*. 2016). Narrative is also employed widely in the education of healthcare professionals (Diekelmann 2001; Ironside 2015; Kawashima 2005; Scheckel and Ironside 2006). The use of narratives in medicine and healthcare, broadly construed, has been variously described as narrative care (Baldwin 2015; Bohlmeijer, Kenyon, and Randall 2010; Mazzoli Smith 2021) or narrative medicine (Charon 2008; Marini 2015; Cenci 2016), where it is said that patients’ explanatory models and their/their families’ notions about illness have enormous clinical significance. For Charon, narrative medicine recognises that the skills that are missing from medicine are in fact narrative competencies, that is, how to systematically adopt others’ points of view, how to recognise the particular along with the universal, how to identify the meaning of individuals’ words, how to enter into an authentic relation with a teller. These tenets of narrative medicine are very influential in wider narrative-based learning in healthcare and were foundational to the development of this framework.

As Walker, Rogers, and Entwistle (2020, 345) state: ‘Many of the claims made about the benefits of attending to narratives overlap with features of person-centred healthcare (PCH). As such, narrative approaches…can be considered potential means of supporting PCH’. At a Consensus Conference in Rome (Conferenza Di Consenso 2015) scholars and professionals drew on 1600 studies in order to define narrative medicine as

…a clinical assistance methodology based on a specific communicative competency. Narrative is the fundamental tool to acquire, understand and integrate the different points of view of those involved in an illness and in the treatment process. (Cenci 2016, 23)

The Caring Stories learning framework draws on this work and in particular the orientation towards narrative as a tool, directed to what is commonly referred to as the fostering of narrative competencies. Reviews of narrative and arts-based medical training initiatives demonstrate the focus is on competencies in the broad arenas of listening and observation skills, communication, empathy, professional growth, increased capacity for reflection, and shared decision-making (Arntfield *et al*. 2013; Barber and Moreno-Leguizamon 2017; Charon and DasGupta 2011; Charon, Wyer, and NEBM Working Group 2008; Haidet *et al.* 2016; Marchalik 2017; Milota, van Thiel, and van Delden 2019; Perry *et al*. 2011; Wieżel *et al*. 2017). Charon suggests the following as likely to drive the process from narrative competence to clinical effectiveness: development of the clinical imagination; deepening of empathy for patients; awareness of the ethical dimensions of clinical situations; the development of the capacity for attention (Charon 2008), also referring to these under the broader moniker ‘narrative competences’ (Charon 2007). Narratives are also said to be important in training to support shared decision-making with patients (Charon and DasGupta 2011; Charon, Wyer, and NEBM Working Group 2008). Others focus on narrative-based training interventions to support the development of listening and observation skills, empathic awareness and an increased capacity for reflection (Marchalik 2017). Teaching programmes and approaches in narrative medicine have in the main focused on these narrative competencies (Arntfield *et al.* 2013).

In literature reviewed by Milota, van Thiel, and van Delden (2019) three basic steps are identified in narrative medicine training approaches; reflective engagement with a patient narrative; corresponding reflection; sharing and discussion in specially constructed environments. However, Wiezel *et al* (2017), Barber and Moreno-Leguizamon (2017) and Fioretti *et al*. (2016) do not find a clear narrative medicine methodology. A systematic review of patients’ illness experience using the narrative medicine approach (Fioretti *et al*. 2016) concluded that narrative medicine has no common specific methodology and call for a definition of the boundaries of the approach when used with patients, in large part in order to then be able to assess outcomes and replicate studies. Fioretti *et al* summarise narrative medicine as follows:

a fundamental tool to acquire, comprehend and integrate the different points of view of all the participants having a role in the illness experience. In this sense, the main aim of the Narrative Medicine approach would be that of co-construct a shared and personalised care path. (Fioretti *et al*. 2016, 8)

We suggest that focusing more clearly on what is meant by narrative as ‘tool’ in the development of a learning framework with these specified outcomes for narrative medicine and PCC contributes towards a common methodology. Goodson and Gill (2014) note that despite the widespread narrative turn, the importance of the ‘pedagogic encounter’ that takes place in everyday settings has been widely disregarded and responding to this, narrative pedagogy brings into sharper focus the learning that is both possible and implicit in the narrative encounter and that which is being sought. The overarching aims of humanisation and personalisation, through increased competency in empathy and listening are broadly agreed on and promoted. However, such broad aims are unlikely to lead to learning without the development of an appropriate pedagogical framework. We suggest and outline below the benefits of situating this learning more rigorously in the literature that problematises core concepts, such as empathy (Macnaughton 2009) and narrative itself (Woods 2011) and an epistemological framework informed by pragmatism.

We take as primarily important for a conceptualisation of narrative as a tool for learning, the caution towards an assumption that through narrative we access an authentic and relatively fixed, or coherent, understanding of the self. There is the concern that healthcare practitioners are not adequately trained to be self-reflexive interpreters of distinctive systems of meaning, as discussed above in relation to narrative learning, but rather naïve realists (Kleinman 2017) and we suggest that how narrative is oriented epistemologically within a learning framework is important in order to promote the former. This stems from debates in philosophy, medical humanities and other disciplines around the ‘narrative turn’ (Czarniawska 2004) and the potential excesses that follow from positioning patients as narrative beings, in an ontological sense, where narrative can come to represent a specific structure of experience and of self-representation (eg, Bruner 2004; Strawson 2004; Woods 2011). Far from narratives expressing everything that is distinctive of an individual patient, the narratives shared by bedsides, in consulting rooms, in the process of undergoing examinations, with carers, and alongside other patients, are the discursive and embodied, partial and contingent communications with a significant other at that point in time. Any meaning derived from these cannot be naively assumed to capture lived experience in a correspondence sense of the truth over time. The usefulness of narrative as a tool for learning, effectively, does not rest on an ontological foundationalism about lived experience and narrative beings.

Narratives are composed for particular audiences at moments in history, and they draw on taken-for-granted discourses and values circulating in a particular culture. Consequently, narratives don’t speak for themselves, offering a window onto an ‘essential self’. (Kohler Riessman 2008)

Following Abettan, it becomes important, heuristically, to make explicit the way in which patients’ narratives are meaningful and the nature of the knowing that is conveyed: ‘The patient’s narrative is meaningful, I can grasp a meaning within it and understand something, however, the knowledge resulting from this understanding is not guaranteed nor constant and may evolve over time’ (2017, 186). It is helpful to conceive of the focus as narrative truth, that is hermeneutical knowledge, or hermeneutical truth, the embedded knowledge of the particular culture, language and society in which one lives. Knowing becomes a form, then, of understanding in context: ‘Hermeneutical knowledge refers to the grasp of a meaning that provides an understanding of what is at stake. It also implies that this meaning evolves over time.’ (2017, 185). Such narrative truth operates in light of—and not as separate from—the epistemological tension that can polarise biomedical, where aligned with positivist approaches to knowledge, and interpretivist, constructivist or critical/postmodern approaches to knowledge generation. Indeed, it is the place and legitimacy of such narrative truth, that pragmatism foregrounds and as such we suggest that it is an important framing for, and pedagogical aid to, narrative-based learning. A pragmatist conception of knowledge, taking Rorty’s (1982, 2009) view, is one that is not based on correspondence to a world of stable facts and so knowledge is not about more or less objective faithful representation to this. Rather, all knowledge is fundamentally ‘constituted’ by a web of meanings (Rorty 1982, 2009). We suggest how this pragmatist orientation can operate pedagogically to support examination of different bodies of knowledge dialogically, rather than in conflict.

Moreno-Leguizamon *et al* (2015) put forward an argument about the need to incorporate and amplify varied epistemologies in the training of health professionals, moving beyond the tendency to talk about ‘two cultures’ and ‘a great divide’ (2015, 19). Their review foregrounds how ‘the contemporary emerging picture is that we know according to differing epistemologies and not just the positivist one with regard to issues of health and illness’ (2015, 19) and as a result there is a clear concern about training which forecloses this. This review is helpful in foregrounding the need not to promote an opposing body of knowledge to the biomedical in narrative-based learning, but a pluralist understanding through ‘a balanced program of education for healthcare professionals’ (2015, 20) and the opportunity to think critically with different ways of knowing. Greenhalgh (1999a) and Greenhalgh and Hurwitz (1999) talk about narratives bridging the gap between mediopathological knowledge and experiential knowledge, and Abettan states that ‘narrative tools and skills cannot be viewed as a method, that is, a knowing process leading to a controlled and verified knowledge' (2017, 188). We demonstrate here how narrative, situated in reflective exercises around the concept of mindset that foregrounds personal epistemological frames, promotes precisely this bridging and epistemological pluralism.

Ways of knowing through narrative is an issue explored in theoretical medicine (Abettan 2017; Alderson 1998; Leder 1990) and medical humanities (Woods 2011), yet one that we would suggest is not adequately brought into focus in discussion of methodologies for training to support PCC and allied aims. Without an epistemic framework orienting learners to these forms of narrative knowing, healthcare training is at risk of implicitly fostering narrative as a method for accessing a fixed understanding of lived experience and/or setting up narrative understanding as in conflict with biomedical knowledge. Baron (1990) highlights this as a concern around the use of narratives in medicine, that is the conceptualising of patients as more static than they are and MacNaugton (2009) points to a danger in assuming we can directly access a patient’s experience. Seeing narratives as a method for accessing lived experience is linked to cutting them off from their epistemological frameworks (Abettan 2017; Baron 1990). Hence we argue for the importance of narrative-based learning in this field that foregrounds epistemology explicitly, which includes pedagogically.

The aim of narrative-based training for PCC should be to support the development of such hermeneutic understanding, rather than the accessing of fixed lived experiences. Yet, pilot work in the UK with social care practitioners found it to be precisely the fixing of someone’s life story through a narrative that was the assumption held about narrative-based training, which also enabled practitioners to maintain stereotypes of service users, found to be a critical barrier to PCC. Such stereotyping prevents development of precisely the kinds of communicative competencies PCC rests on. Walker *et al* offer another important articulation of why narrative modes of knowing should be thought of as pluralist:

Since our pluralist conception also implies the knowledge gained from narratives is continuous with other knowledge…'listening to narratives’ is not some silver bullet for achieving a current understanding, but rather encourages critical thinking about how both clinician and patient are interpreting events and assessment of interpretation in light of other knowledge. (Walker, Rogers, and Entwistle 2020, 4)

American pragmatism (Dewey 1986; Rorty 1982) lends itself as an ontological and epistemological framework for such a pluralist conception of narrative and we show how there are also important pedagogical implications (Mazzoli Smith 2021). The learning framework of Caring Stories is innovative because of this explicit use of pragmatist pluralism. By pluralism in the pragmatist sense, we are referring to the view espoused by Dewey and Rorty, that the search for a unified and coherent ‘truth’ is really about arriving at a provisional agreement about differing accounts of what is the case. Philosophically pragmatism need not be intrinsically allied with pluralism (Misak 2005), but on this view of pragmatism (Dewey 1910/2012; Rorty 1982), epistemological pluralism holds that we cannot arrive at a final, commensurate state of knowledge. The importance for narrative-based learning here is that this implies the need to engage with differing forms or knowledge and attendant norms, which in turn invokes dialogue as a necessary vehicle for learning. We provide an overview of the Caring Stories Learning Framework below to explicate how these issues have informed its methodology and pedagogy.

## The Caring Stories Learning Framework

…it is extended practice with narratives that makes us capable of appropriately handling and understanding multiple perspectives and attitudes of particular events…By developing our narrative understanding through such practice we become sensitive to a variety of possible perspectives that may be adopted on events, including – especially – cognitive, emotional and evaluative perspectives that may diverge from our own. (Hutto and McGivern 2016)

As a digital learning platform, Caring Stories takes advantage of and speaks to the ways in which new technologies and social media promote the sharing of narratives around healthcare and illness (Cenci 2016). Patients and older members of the public were involved in the design of the learning platform in a series of co-production workshops from the outset, which were both focused on generating narratives that we could build into the training materials, but also in respect of co-designing aspects of the training as it was developed. We worked with key older patient experience groups and institutions in four countries, the UK, the Netherlands, Spain and France, in the development of the training platform: VOICE at the National Innovation Centre for Ageing, Newcastle University; Leiden Academy on Vitality and Ageing; the Hospital Clínic de Barcelona, a public consortium and community hospital made up of the Government of Catalonia (CatSalut) and the University of Barcelona; and the EU project ESeniors, based in Paris.

The pedagogical framework of Caring Stories is devised around a series of exercises that interrogate the various aspects of narrative that constitute its utility as a tool. Certainly the sense in which meaning is conveyed as content that is heard is one part of this, but also the sense in which the narrative encounter is itself what situates such meaning. The challenge of such a narrative-based pedagogy is in the need to build this particular, nuanced, hermeneutic understanding experientially. Such experiential learning depends on the capacity of the learner to reflect on their narratives as similarly situated. We focus on the importance of *interpreting* narratives at all stages of the training, the point here being that narratives are already always being interpreted, on some level and to some extent, if shared.

Small group sessions by skilled facilitators were found to be key in the review of narrative medicine education by Barber and Moreno-Leguizamon (2017) and are used to good effect in other structured and powerful reflective learning methods, such as digital story-telling (Lambert 2013). The requirements for the training are therefore an environment conducive for dialogue and the narrative resources for the exercises, either provided, or generated by trainees in the first foundational module. The logic underlying the progression of the modules is the building of a nested, composite web of inroads into different ways of interpreting narrative and in bringing to the fore different structural components of narrative, inspired by the idea of a composite approach to narrative inquiry (Brown *et al*. 1989). The overarching learning aim can be said to be the development of the disposition towards interpretation, drawing on and deepening competencies around listening, communicating and contextualising narrative knowledge. The key learning process through which this is fostered is reflective learning, or reflective knowledge-in-action (Schön 1983).

### Module 0: Story reading/generating

This first module is foundational, designed to orient learners to the value of narratives in healthcare, introducing what we mean by narrative and key concepts and ideas in narrative-based learning. By working with the idea of what a narrative is and how we might actually think with narratives, the training makes explicit aspects of the discussion above. It does so primarily through exercises which both call on learners to generate narratives about aspects of their lives, as well as to generate narratives with patients, residents or stakeholders in their organisations. Even what might be considered to be the self-evident process of engaging someone so as to elicit a narrative is itself part of a training informed by the methodology of narrative inquiry. The exercises in this first module link to a protocol that was developed through engagement with a broad literature on narrative inquiry and narrative interviewing. Crucial here is the fact that;

When the research interview is viewed as a conversation – a discourse between speakers – rules of everyday conversation will apply: turn-taking, relevance, and entrance and exit talk…One story can lead to another, as narrator and questioner/listener negotiate openings for extended turns and associative shifts in topic. (Kohler Riessman 2008, 24)

Engaging learners in some of the tenets of narrative-based research may never have been a part of their experience, despite the fact that they will have encountered and engaged with many patient narratives and so the exercises focus on some of these core narrative research skills through use of the protocol to support generating narratives. This first module promotes a disposition towards narrative that we argue is generative of the kind of learning that foregrounds how narratives function as communicative acts, rather than fixed conveyors of meaning.

### Module 1: Mindset orientation and reflection

This module introduces trainees to the concept of mindset, understood here as the lens through which we habitually see and respond to the world. Rydén, Ringberg, and Wilke (2015) describe mindset as the mental models that we hold, which are both private and cultural and underpin individual sense-making. PCC is also introduced as the key concern of the module, which draws on reflective learning to help trainees consider their particular mindset in relation to the benefits and challenges of PCC. For instance, the mindset orientation of learners holding to the biomedical model can appear, in interaction, to be at odds with the constructivist knowledge of narrative cognition, that which is primarily the realm of the patients. Use of the ‘mindset’ concept in exercises in this module necessitates explicit reflection on these epistemic issues. Thinking about healthcare in dualistic terms; medical/psychosocial history (Wear and Castellani 2000), or the universal/particular, the positivist/constructivist and so on, can be both unhelpful but also misrepresentative of the complexity of healthcare practices (Moreno Leguizamon, Patterson, and Rivadeneira 2015).

Pragmatism enables a reorientation away from questions of truth, to one of interpretations that can produce new and better ways of thinking and acting in light of situated interaction. Within a pragmatist framing, narrative interpretation is not infinite and unbounded and dialogical exercises at this stage work with the concept of criteria that can be brought directly into conversation with each other to offer foundations for situated judgement and interpretation. For the adult professional learner in healthcare, the focus here is towards the usefulness of incorporating more and therefore potentially better forms of interpretation into the situated healthcare encounter. We suggest that making this pluralist conception of knowledge explicit in the learning is important to mitigate the tendency for learners to defend themselves against the easy slide into the language of conflictual epistemological stances, as Moreno Leguizamon *et al* caution against. One way we do this is by using Patient Voices digital stories in exercises, asking trainees to explore complex and multiple roles and perspectives, and also through reflective journalling.2

This scaffolding of interpretation at a meta level fosters a disposition and motivation towards cognizant thinking, another concept used in the training, along with categorical thinking. Here categorical thinking characterises routine performance and working under pressure, being more resistant to change, while cognizant, or reflective thinking, denotes a high level of cognitive responsiveness and flexibility (Ringberg and Reihlen 2008). In the training we situate cognizant thinking as central to a disposition that supports interpretative competencies. Rydén, Ringberg, and Wilke (2015) discuss how a shift from categorical to cognizant thinking depends on the reflexivity to sense dissonance between external feedback and the learners’ own sense-making, through dialogue and in particular, questioning, as used in the module exercises. This kind of flexibility and sense-making is necessary to respond to what Macnaughton describes:

Clinicians atomise their patients (psychologically and physically) but at the same time are expected to relate to them as complete entities, or essences. This can require many shifts in perspective during the course of a single consultation. (Macnaughton 2009, 1940)

### Module 2: Content-based story interpretation

This module looks at the content of stories and exercises explore how healthcare professionals can better think with narrative content and interpret it, specifically in the context of limited time and specific pressures in professional roles. The key focus of the exercises is to situate content-based interpretation around a core tenet of narrative analysis, such that ‘extended accounts are preserved and treated analytically as units, rather than fragmented into thematic categories’ (Kohler Riessman 2008, 12). Exercises include distillation to core stories, or the mapping of storylines and events visually.

For Gadamer (1975), in order to pay attention to the meaning implicit in a narrative and achieve understanding, the criterion of questioning, implicit or explicit, external or internal, is therefore very important. The hermeneutic task becomes a questioning of things, based on the hermeneutic disposition that we must adopt when meaning is not immediately obvious, such as when engaged in dialogue or reading texts. A dialogical approach is therefore key to the exercises in the training, as questions are posed within the dialogical group about interpretations of content drawn in the presence of the other, which fosters understanding as practical and situated, foregrounding the web of meanings and contexts in which understanding necessarily takes place (Goodson and Gill 2014).

### Module 3: Structure-based story interpretation

The exercises in this training module focus on the formal, structural features of narrated stories, which are foregrounded as a heuristic device to foster deeper skills in interpretation. Structural features of stories add to the content with respect to how the narrator achieves their persuasive aims. Engaging explicitly with such features supports healthcare practitioners to better interpret the emotional and imaginative subtexts of their patients’ stories. As Kohler Riessman says ‘These questions shift attention from the ‘told’ to the ‘telling’ and from exclusive focus on a narrator’s experience to the narrative itself’ (2008, 77). We suggest the value of exercises that foreground the structural features of stories is based on how much less immediately apprehensible such features are, than considerations of content. In everyday engagement we tend to foreground the content of stories, or at least our awareness of content is in the foreground, over considerations of structural features, or rhetorical elements. Yet these aspects are integral to how we hear and therefore interpret meaning. Exercises ask learners to analyse textual features such as metaphors in stories, for instance. Metaphors draw from, and hence reference, sociocultural discourse and are resonant of the complex interdependencies of experience, facilitating the expression of complexity (Martin 2010).

Exercises in this module also draw on narrative pedagogy (Goodson 2012), categorising life stories along a broad spectrum from ‘description’, referring often to chronological series of largely factual, retrospective descriptions, to ‘elaboration’, where the story is theorised, analysed and reflected on. These are not presented as either fixed or absolute types and Goodson theorises a complex relationship between narrative categories and agency. With a pedagogical aim in mind, asking trainees to engage with this categorisation of story structures is useful as a heuristic to aid reflection and interpretive work at this structural and less visible level of narrative. Patient Voices digital stories are again an excellent resource here as the multimodal form including pictures and music supports learners to engage with structural aspects of stories such as form and genre.

### Module 4: Integrated story interpretation

This module looks at how the narratives of different people, roles and organisations come together in the healthcare of an individual. Patients engage with many different healthcare professionals and professionals work with other professionals in different roles, creating synergies and/or tensions. The module is focused on the importance of learning across these narratives, at the institutional level and the importance of dialogue in negotiating these. Learning design draws on Engeström (2015), itself developed from cultural-historical activity theory (Vygotsky and Cole 1978) and Bateson (2000). The multivoicedness of activity systems creates different positions for participants carrying their own diverse histories, with the activity system (eg, a hospital) and ‘multiple layers and strands of history engraved in its artifacts, rules, and conventions’ (Engeström 2018, 49). The narrative-based training explicitly brings together intrainstitutional and interinstitutional stories.

The module exercises are informed by Engeström’s boundary-crossing exercises, expansive learning processes focused on interconnected activity systems with contradictory demands across them. Learning across the systems then depends on learning from patients/professionals/families in each, to better understand care relationships and critical paths that could provide solutions to perceived contradictions. ‘An expansive transformation is accomplished when the object and motive of the activity are reconceptualized to embrace a radically wider horizon of possibilities than in the previous mode of activity’ (Engeström 2018, 50). So learning here cannot be based on individual stories alone, because the issue at stake is organisational. Exercises ask trainees to bring to the fore the differing stances, perspectives and drivers situated in different roles through activities that juxtapose and analyse these in taking different perspectives. We suggest that the fostering of such wider horizons of possibilities is supported by and largely predicated on learners’ prior work on mindset and with hermeneutic analysis in general.

### Module 5: Practice-based skills

The final module is focused on embedding learning situated around dialogical groups into the context of the learners’ own professional context. This stage in particular draws on transformative learning theory, understood as the process of using a prior interpretation to construe a new or revised interpretation of the meaning of one’s experience in order to guide future action (Taylor 1998). The focus in this approach on how our expectations, framed within cultural assumptions, directly influence the meaning we derive from our experiences, has a clear link to the interpretative competencies the training aims to develop through narrative-based interpretation. It is then perspective transformation, or the move to cognizant thinking, that entails a revision of meaning structures, which is transformative (Taylor 1998).

Mezirow describes perspective transformation as ‘a more fully developed (more functional) frame of reference’ (1996, 163), that is more inclusive, differentiating, permeable, critically reflective and integrative of experience. Transformation in this sense of learning involves some degree of alienation from earlier established conceptions of values and one’s action in the world, and the reframing of new perspectives. Critical self-reflection at a cognitive level is seen to be the key to adult transformative learning theory, as is meaning-making, as transformative learning can be ‘understood as a continuous effort to negotiate contested meanings’ (Mezirow 2000, 3).

Adult transformative learning is predicated on a process of sense-making and interpreting experience through heightened awareness and understanding, which can be defined in pedagogical terms as re-examining assumptions, synthesising and justifying and thereby acting on new meaning perspectives. Critical reflection has a very specific meaning in transformative learning and hence adult education, based on Habermas (2015), that is awareness of being caught in one’s own history and learning to question the integrity of assumptions and beliefs based on past experiences (Mezirow 1996). Narrative is used as the tool through which to reveal and explore assumptions, shaped in dialogical questioning and shifts in perspective from earlier modules are explicitly brought to the fore and become the focus of dialogical exercises.

Transformative learning is occasionally referenced as part of medical education programmes in relation to the change that a student medic needs to go through on the journey to clinician (Abela 2009; Greenhill and Poncelet 2013). Learning objectives including ‘critical thinking, clinician scepticism, and systems thinking in the clinical curriculum to promote perspective change’ (Greenhill and Poncelet 2013) are advocated and conceptualised through Mezirow’s transformative learning theory. It is also a framework used to understand the training of non-technical skills (Kerins *et al.* 2020). However, it is not a central concept to learning for PCC more broadly construed, although it could strongly support it we suggest, if integrated with the particular approach to narrative that we explicate.

## Conclusion

Britten *et al*. (2020) in discussing the Gothenburg Model of PCC, state that there is no information available about how to learn about PCC and we would suggest that in part this reflects an absence of pedagogical theory in the literature. The learning framework outlined in this paper is aimed at fostering practitioners’ awareness and increased engagement of the meaning frames of the patients they care for, through increased interpretive and communicative competencies, but predicated on a foundation of engagement in their own personal meaning frames and epistemological standpoints, through increased critical reflexivity. We suggest that orienting the training within a pragmatist conception of knowledge serves not only a useful epistemological purpose through which to counter dualist and foundational thinking, which can lead to resistance towards narrative medicine and person-centred training, but also functions as a pedagogical resource to foster experiential learning. The essence of transformative learning comes through critical self-reflection and active meaning-making with three main elements; critical reflection on assumptions, dialectical discourse to validate new meaning perspectives; and the context of the learning experience (Greenhill *et al*. 2018; Mezirow 2000). We suggest that these steps are apposite to the core aims of training focused on PCC and narrative competencies and the Caring Stories learning framework provides an explicit framework through which this can occur.

It is not assumed to be the case that implementing this training is straightforward. Pilot work has demonstrated that the orientation of the trainer is critical. Valuing and fostering inclusivity to enable the sharing of heterogeneous views in dialogue is essential to make the most of the learning methodology and exercises. Time limitations for this kind of training are often an issue, as is the need to produce an evaluation of short-term impact and outcomes, which can be far from transparent in a causal and linear sense. In the Netherlands pilot work also suggests that professionals are not keen to share their stories or perspectives on patient stories in an organisational culture in which they do not feel safe. Short initial individual meetings with trainees are being explored in order for trainers to better understand possible constraints of the organisational culture and also more detailed trainee needs. Piloting is also exploring how the methodology can itself support the co-design of context-specific organisational goals or aims towards PCC.

This paper has described a learning methodology for how the plurality of pragmatist epistemology, along with a foundational relational ontology, situates the relationship of the practitioner/patient context as central and narrative as a relational and communicative tool in context, rather than as a method for uncovering the fixed patient experience or identity. This is important as we would suggest that trainings which do not make these aspects explicit in both methodology and pedagogy implicitly draw on an epistemic understanding likely to be influenced, at least to some extent, by positivism, with an assumption of atomised individuals, with capacities for caring that somehow reside individually within them, in a largely decontextualised way. Narratives are then taken as objectified patient knowledge, disembodied, detached and fixed. A pragmatist framing suggests there cannot be anything other than epistemic plurality, considered in action, shifting the focus of attention for learning from individuals and a foundationalist understanding of truth, towards the experiential and relational of the fluid nature of care.

## Data Availability

Data sharing is not applicable as no data sets were generated and/or analysed for this study.

## Notes

1. The Caring Stories training platform was funded by EIT Health grant number RF230156 and is available at: http://www.caring stories.eu.

2. Patient Voices is a repository of more than 1000 digital stories of health and healthcare: http://www.patientvoices.org.uk/. Digital storytelling is a reflective process that scaffolds the creation of short, multi-modal stories in video format (Lambert 2013).
